# A TV–BM3D Iterative Algorithm for VMAT-CT Reconstruction

**DOI:** 10.3390/jimaging12040166

**Published:** 2026-04-10

**Authors:** Chia-Lung Chien, Beibei Guo, Rui Zhang

**Affiliations:** 1 Department of Radiation Oncology, St. Jude Children’s Research Hospital, Memphis, TN 38105, USA; chialung.chien@stjude.org; 2 Department of Experimental Statistics, Louisiana State University, Baton Rouge, LA 70803, USA; beibeiguo@lsu.edu; 3 Department of Radiation Oncology, Baylor College of Medicine, Houston, TX 77030, USA; 4 Department of Physics and Astronomy, Louisiana State University, Baton Rouge, LA 70803, USA

**Keywords:** volumetric modulated arc therapy, computed tomography, iterative reconstruction, compressed sensing, total variation, block-matching and 3D filtering

## Abstract

Volumetric modulated arc therapy-computed tomography (VMAT-CT), which is the CT reconstructed using the portal images collected during VMAT, can potentially be an effective onsite imaging tool. The goal of this study was to propose an iterative reconstruction algorithm that can further improve the image quality of VMAT-CT and reduce the number of failed reconstructions. An iterative algorithm combining total variation (TV) with block-matching and 3D filtering (BM3D) was proposed, addressing the L1-L2 regularization problem using the split Bregman method. We collected portal images from 67 VMAT cases including 50 phantom and 17 real-patient cases. Both Feldkamp–Davis–Kress (FDK) and TV-BM3D iterative algorithms were used to reconstruct VMAT-CT using the collected images. The preprocessing methods developed by our group previously were also used in this study. A total of 48 out of 50 phantom cases and 15 out of 17 real-patient cases were successfully reconstructed using the iterative algorithm together with image preprocessing. In contrast, 39 phantom cases and 8 patient cases could be reconstructed using the original FDK algorithm, and 44 phantom cases and 11 patient cases could be reconstructed using the FDK algorithm together with preprocessing. Compared with the FDK algorithm, the TV-BM3D iterative algorithm significantly improved the image quality of VMAT-CT at all treatment sites. To the best of our knowledge, this study is the first to develop an iterative VMAT-CT reconstruction algorithm. It can be used to reconstruct CT images locally, and is superior to FDK-based algorithms in terms of the success rate and reconstructed image quality. This strongly supports the use of VMAT-CT as a promising imaging tool for treatment monitoring and adaptive radiotherapy.

## 1. Introduction

Volumetric modulated arc therapy (VMAT) is a popular rotational radiotherapy (RT) technique due to its faster delivery, increased degree of freedom for dose optimization, and improved dose conformity [1,2]. To detect the intra-fractional anatomical changes without introducing extra imaging dose or cost, Poludniowski et al. [3] proposed to reconstruct megavoltage (MV) computed tomography (CT) using electronic portal imaging device (EPID) images collected during VMAT and named it VMAT-CT. The proposed reconstruction is a three-dimensional (3D) lambda tomography (LT) method based on the Feldkamp–Davis–Kress (FDK) algorithm and lambda filter [3]. However, the poor image quality of VMAT-CT due to data insufficiency, truncation and blurriness hindered its applications in clinic. To improve the image quality of VMAT-CT, our group proposed a new extrapolation scheme that extrapolates along collimator angles instead of horizontal direction to preserve most of the useful information in EPID images [4]. Furthermore, we proposed systematic methods to preprocess EPID images, including online region-based active contouring, multi-leaf collimator (MLC) motion modeling, and outlier filtering, and significantly improved the image quality of VMAT-CT for multiple treatment sites (head and neck, lung, and esophagus) [5].

However, our methods still failed to reconstruct VMAT-CT from certain VMAT plans that had extremely insufficient projection data. The failures resulted from the inherent limitation of the LT algorithm: the reconstruction quality degrades dramatically if the projection sampling is sparse or the projection angle range is less than 180° plus the fan angle [6,7]. Although the LT is non-quantitative in that it does not require an exact and unique reconstruction [8,9], the reconstruct may fail if the sampled angle range for certain voxels is fewer than the lowest acceptable cutoff threshold (1.57 radians or 90°) [3].

Iterative reconstruction algorithms for incomplete projection data have been proposed to overcome the limitations of the LT algorithm. The iterative algorithms can introduce image constraints [10,11,12,13], which can be prior known information or realistic assumptions of the missing data such as positivity of voxel values, bounds of image smoothness and voxel values, so the reconstruction can be protected from unrealistic artifacts and distortions coming from data deficiency.

The concept of compressed sensing (CS) was proposed in 2006 [14]. According to CS theory, a signal can be recovered from fewer samples than the number required by Nyquist sampling theorem if the signal is sparse. Consequently, if an image f can be sparsified by operations such as a discrete gradient operator [15,16], the image can be reconstructed from less sampling. Sidky et al. proposed an iterative algorithm for CT reconstruction based on CS theory and incorporated total variation (TV) minimization [17], and later developed the iterative algorithm named adaptive-steepest-descent-projection-onto-convex-sets (ASD-POCS) [18]. Since then, other studies have adopted TV minimization in the iterative reconstruction to solve the problem of insufficient projection data [19,20,21].

TV minimization has an assumption that the pixel values within the structures in a CT image are piecewise constant such that the non-zero signal concentrates on the boundaries of these structures in the TV domain of the CT image [22,23]. Under this assumption, TV minimization can exploit the gradient sparsity by the L1 regularization technique, protect the edges of internal structures within images, and smooth out noises within the anatomical structures, which is suitable for medical images that have a uniform intensity within a structure. However, if the image intensity fluctuates drastically because of complicated structures or large streaking artifacts, TV minimization might introduce staircase artifacts that degrade the image quality and fail to remove the streaking artifacts in the CT reconstruction [24]. Although many revisions of TV minimization have been proposed to solve this problem such as edge-preserving TV [25], anisotropic TV [26], and adaptive-weighted TV [27], they are still not effective for images with poor image contrast and require considerable tuning labors.

The block-matching and 3D filtering (BM3D) method [28,29] is an advanced image-denoising method that can also encourage data sparsity. In this method, 3D stacks, which are similar patches grouped by block-matching, can be sparsified by linear transform such as Fourier transform and hard-thresholding such as L1 regularization [28]. While TV minimization utilizes gradient sparsity in the spatial domain, the BM3D method achieves sparse representation in the transform domain but with the assumption that an anatomical structure recognized by blocking matching would have a similar appearance throughout a medical image. Unlike TV minimization, the BM3D method does not expect an image to have uniform intensity within a structure, thus avoiding the introduction of staircase artifacts when the image intensity fluctuates. Therefore, several groups have proposed iterative algorithms for CT reconstruction using BM3D filters [30,31,32].

The goal of this study was to develop a CS-based iterative algorithm for VMAT-CT reconstruction. This algorithm utilizes both TV minimization and BM3D denoising, and can further improve the image quality of VMAT. The preprocessing methods previously developed by our group [5] were also used in this study to achieve the best results. To the best of our knowledge, this study developed the first iterative VMAT-CT reconstruction algorithm that is superior to FDK-based algorithms in terms of success rate and reconstructed image quality.

## 2. Materials and Methods

### 2.1. TV-BM3D Iterative VMAT-CT Reconstruction

The TV of a 3D CT image f was defined as the sum of the L1 norms of its discrete gradient in the *x*, *y*, and *z* directions at every voxel:

(1) $${\left\|f\right\|}_{\mathrm{TV}}={\left\|\nabla_{x}f\right\|}_{1}+{\left\|\nabla_{y}f\right\|}_{1}+{\left\|\nabla_{z}f\right\|}_{1}$$

In the traditional algorithm, TV minimization was incorporated into the iterative algorithm to solve the following convex optimization problem:

(2) $$f^{*}=\underset{f}{\min}\left\{{\left\|f\right\|}_{\mathrm{TV}}+{\mu \left\|Rf-p\right\|}_{2}^{2}\right\}$$

where R is the forward projection operator, p is the raw projection data, *μ* is the hyperparameter controlling the weight of the regularization, and ${\left|\left|f\right|\right|}_{TV}$ is the TV regularization term shown above and represents the sparseness constraint of the CT image. The traditional algorithm treats the optimization as two phases in each iteration: the first phase is to enforce the projection data consistency, which is represented by the fidelity term (${\left||\;Rf-p|\right|}_{2}^{2})$, using the simultaneous algebraic reconstruction technique (SART) [33] and the non-negativity of the reconstructed CT (f); the second phase is to minimize TV with the adaptive steepest gradient descent algorithm [10].

**Figure 1:**
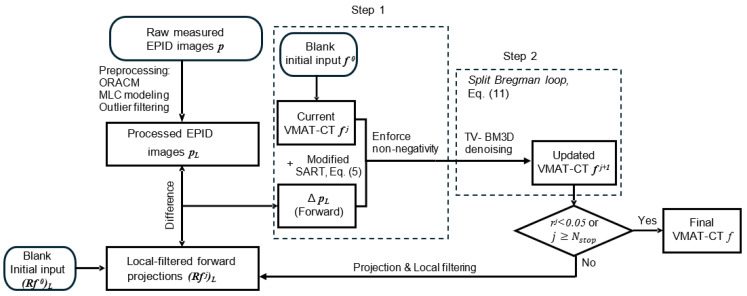
The flow chart of the iterative TV-BM3D reconstruction algorithm proposed in this study. ORACM: Online region-based active contouring; MLC: Multi-leaf collimator.

However, because VMAT-CT reconstruction is performed within a local volume that is much smaller than the field of view of open-field conventional CT or cone beam CT (CBCT), the projection operation Rf, which represents the Radon transform in discrete form, fails to describe the incomplete Radon transform situation of VMAT-CT. Instead, the projection operator should be modified with the local filtering operator, which would work effectively for projection data truncation [34,35,36]. Therefore, we modified the fidelity term to be ${\left|\left|{\left(Rf\right)}_{L}-{\left(p\right)}_{L}\right|\right|}_{2}^{2}$ to relate the projection operation of VMAT-CT to the raw truncated EPID images, where *L* represents the local filtering. Furthermore, we incorporated the BM3D regularization term besides TV regularization into our TV-BM3D iterative algorithm. The final optimization problem could be expressed as

(3) $$f^{*}=\underset{f}{\min}\left\{{\left\|f\right\|}_{\mathrm{TV}}+{\mu \left\|(R{f)}_{L}-p_{L}\right\|}_{2}^{2}+\delta BM3D\left(f\right)\right\}$$

where $BM3D\left(f\right)$ is the BM3D regularization term [37,38], *δ* is the hyperparameter controlling the weight of BM3D regularization and is set to 1, and the EPID image after local filtering operation can be represented as

(4) $$p_{L}\left(u,v\right)=p\left(u,v\right)⊗e_{R}\left(u\right)$$

where (*u*, *v*) is the generic EPID coordinate and $e_{R}$ the convolution kernel defined by the local filter [3]. Figure 1 shows the flow chart of the iterative TV-BM3D reconstruction algorithm proposed in this study.

Considering it is tedious to iteratively solve the L2 fidelity term and the two L1 regularization terms of Equation (3), we adopted the split Bregman iteration method and a two-step iteration to transform the difficult L1–L2 problem into a sequence of subproblems and Bregman updates [38,39,40,41,42].

The first step is to minimize the fidelity term

(5) $$f^{j}=\underset{\tilde{f}}{\min}{\left\|(R{\tilde{f})}_{L}-p_{L}\right\|}_{2}^{2}$$

After the raw EPID data were preprocessed [5], the projection difference between the filtered EPID images and the locally filtered forward projections from the current VMAT-CT (a blank input initially) was calculated as $\Delta p_{L}=p_{L}-(R{f)}_{L}$. This projection difference $\left(\Delta p_{L}\right)$ was then back-projected to the VMAT-CT domain to generate Δ*f_L_* using a modified SART [3,35,36,43], and the VMAT-CT $\left(f\right)$ (a blank CT initially) was updated accordingly. The modified SART operates only within the localized volume of interest and is expressed as
Modified SART$x_{1}=f^{j}$;**for**$\;i=1:N_{\theta }$$\;\;\;\;\;\;\;x_{i+1}=x_{i}+∆f_{L}$$=x_{i}+I\cdot \lambda V_{\theta_{i}}^{-1}R_{\theta_{i}}^{T}W_{\theta_{i}}{\left(\Delta p_{L}\right)}_{\theta_{i}}\;\mathrm{such}\;\mathrm{that}\;\;{\left(\Delta p_{L}\right)}_{\theta_{i}}\in M_{\theta_{i}}$;**end**$updated\;f^{j}=x_{N_{\theta }+1}$;

Here, $f^{j}$ and $updated\;f^{j}$ are VMAT-CT before and after the modified SART update, N_θ_ is the number of gantry angles, *R* is the forward projection operator, *V* is the diagonal matrix with nth diagonal element as $V_{nn}=\sum_{m\in M_{\beta }}\left|R_{mn}\right|$, *W* is the diagonal matrix with *m*th diagonal element as $W_{mm}=\frac{1}{\sum_{n\in I_{VOI}}\left|R_{mn}\right|}$, *λ* is the hyperparameter for iterations, I is the 3D masking function for the reconstruction volume based on planning target volume, $M_{\theta }$ is the two-dimensional masking function of each EPID image at gantry angle θ. We also enforced the non-negativity of each voxel in the VMAT-CT reconstruction after each modified SART update.

The second step is the TV-BM3D denoising based on the split Bregman method. After the first step of minimization of the fidelity term, the optimization problem of Equation (3) can be transformed and written as [38,39,40]

(6) $$\begin{array}{c} \underset{\tilde{f}}{min}\left\{{\left\|D_{x}\right\|}_{1}+{\left\|D_{y}\right\|}_{1}+{\left\|D_{z}\right\|}_{1}+\mu {\left\|\tilde{f}-f^{\mathrm{it}}\right\|}_{2}^{2}+\delta \left|D_{w}\right|\right\}\\ \mathrm{such}\;\mathrm{that}\;D_{x}=\nabla_{x}\hat{f},{\;D}_{y}=\nabla_{y}\hat{f},\;\;\;\;\;\;D_{z}=\nabla_{z}\hat{f},\;\;\;\;\;\;D_{w}=BM3D\left(\hat{f}\right) \end{array}$$

By applying the Bregman iteration with multiple penalty terms, the constrained problem can be fulfilled as

(7) $$\begin{array}{cc} {\hat{f}}^{k+1},\;D_{x}^{k+1},\;D_{y}^{k+1}, & \;D_{z}^{k},\;D_{w}^{k+1}\\ & =\underset{\hat{f},\;D_{x},\;D_{y},\;D_{z},\;\;D_{w}}{min}\{{\left\|D_{x}\right\|}_{1}+{\left\|D_{y}\right\|}_{1}+{\left\|D_{z}\right\|}_{1}+{\mu \left\|\hat{f}-f^{j}\right\|}_{2}^{2}\\ & +\delta {\left\|D_{w}\right\|}_{1}+\alpha {\left\|D_{x}^{k}-\nabla_{x}\hat{f}-b_{x}^{k}\right\|}_{2}^{2}+\alpha {\left\|D_{y}^{k}-\nabla_{y}\hat{f}-b_{y}^{k}\right\|}_{2}^{2}\\ & +\alpha {\left\|D_{z}^{k}-\nabla_{z}\hat{f}-b_{z}^{k}\right\|}_{2}^{2}+{\beta \left\|D_{w}^{k}-BM3D\left(\hat{f}\right)-b_{w}^{k}\right\|}_{2}^{2}\} \end{array}$$

where k represents the *k*th denoising loop; *α*, and *β* are denoising parameters to tune the accuracy of $D_{x},\;D_{y},\;D_{z},$ and $D_{w}$, respectively; $b_{i}^{k}$ is given by the split Bregman iteration.

The split Bregman method could solve the pluralistic problem by successively minimizing L1 and L2 components with respect to $\hat{f}$, $D_{x},\;D_{y},D_{z},\;\mathrm{and}\;D_{w}$:

(8) $$\begin{array}{c} {\hat{f}}^{k+1}=\underset{\hat{f}}{min}\{{\mu \left\|\hat{f}-f^{j}\right\|}_{2}^{2}+\alpha {\left\|D_{x}^{k}-\nabla_{x}\hat{f}-b_{x}^{k}\right\|}_{2}^{2}+\;\alpha {\left\|D_{y}^{k}-\nabla_{y}\hat{f}-b_{y}^{k}\right\|}_{2}^{2}\\ +\alpha {\left\|D_{z}^{k}-\nabla_{z}\hat{f}-b_{z}^{k}\right\|}_{2}^{2}+{\beta \left\|D_{w}^{k}-\mathrm{BM}3D\left(\hat{f}\right)-b_{w}^{k}\right\|}_{2}^{2}\} \end{array}$$

and

(9) $$\left\{\begin{array}{l} {D_{x}^{k+1}=\underset{D_{x}}{min}\left\|D_{x}\right\|}_{1}+\alpha {\left\|D_{x}^{k}-\nabla_{x}{\hat{f}}^{k+1}-b_{x}^{k}\right\|}_{2}^{2}\\ \begin{array}{l} {D_{y}^{k+1}=\underset{D_{y}}{min}\left\|D_{y}\right\|}_{1}+\alpha {\left\|D_{y}^{k}-\nabla_{y}{\hat{f}}^{k+1}-b_{y}^{k}\right\|}_{2}^{2}\\ \begin{array}{l} {D_{z}^{k+1}=\underset{D_{z}}{min}\left\|D_{z}\right\|}_{1}+\alpha {\left\|D_{z}^{k}-\nabla_{z}{\hat{f}}^{k+1}-b_{z}^{k}\right\|}_{2}^{2}\\ {D_{w}^{k+1}=\underset{D_{w}}{min}\left\|D_{w}\right\|}_{1}+{\beta \left\|D_{w}^{k}-\mathrm{BM}3D\left({\hat{f}}^{k+1}\right)-b_{w}^{k}\right\|}_{2}^{2} \end{array} \end{array} \end{array}\right.$$

where the values of $b_{x}^{k},\;b_{y}^{k},b_{z}^{k},{\mathrm{and}\;b}_{w}^{k}$ can be solved as

(10) $$\;\left\{\begin{array}{l} b_{x}^{k+1}=b_{x}^{k}+\nabla_{x}{\hat{f}}^{k+1}-D_{x}^{k+1}\\ \begin{array}{l} b_{y}^{k+1}=b_{y}^{k}+\nabla_{y}{\hat{f}}^{k+1}-D_{y}^{k+1}\\ \begin{array}{l} b_{z}^{k+1}=b_{z}^{k}+\nabla_{z}{\hat{f}}^{k+1}-D_{z}^{k+1}\\ b_{w}^{k+1}=b_{w}^{k}+BM3D\left({\hat{f}}^{k+1}\right)-D_{w}^{k+1} \end{array} \end{array} \end{array}\right.$$

when the variables ${\hat{f}}^{k+1},D_{x}^{k+1},\;D_{y}^{k+1},\;D_{z}^{k+1},D_{w}^{k+1}$ are fixed.

Since ${\hat{f}}^{k}$ is decoupled from the L1 components of the problem, the solution of ${\hat{f}}^{k}$ could be achieved by Fourier transform method and expressed as [39]

(11) $${\hat{f}}^{k+1}=\mathrm{ifft}2\left\{\frac{\mathrm{fft}2\left(\mu f^{j}+\alpha \left(\nabla_{x}^{T}\left(D_{x}^{k}-b_{x}^{k}\right)+\nabla_{y}^{T}\left(D_{y}^{k}-b_{y}^{k}\right)+\nabla_{z}^{T}\left(D_{z}^{k}-b_{z}^{k}\right)\right)+\beta \left(D_{w}^{k}-b_{w}^{k}\right)\right)}{\mathrm{fft}2\left(\mu +\alpha ∆+\beta \right)}\right\}$$

where *fft2* and *ifft2* represent 2D Fourier transform and inverse Fourier transform; Δ is the Laplace operator; $\nabla_{x}^{T},\;\nabla_{y}^{T},\;\mathrm{and}\;\nabla_{z}^{T}$ are the transpose gradient operators [38].

Also, the solutions of Equation (9) are given using the shrinkage operator:

(12) $$\left\{\begin{array}{l} D_{x}^{k+1}=\mathrm{shrink}\left(\nabla_{x}{\hat{f}}^{k+1}+b_{x}^{k},\frac{1}{\alpha }\right)\\ D_{y}^{k+1}=\mathrm{shrink}\left(\nabla_{y}{\hat{f}}^{k+1}+b_{y}^{k},\frac{1}{\alpha }\right)\\ D_{z}^{k+1}=\mathrm{shrink}\left(\nabla_{z}{\hat{f}}^{k+1}+b_{z}^{k},\frac{1}{\alpha }\right)\\ D_{w}^{k+1}=\mathrm{shrink}\left(\mathrm{BM}3D\left({\hat{f}}^{k+1}\right)+b_{w}^{k},\frac{1}{\beta }\right) \end{array}\right.$$

where the shrink function is defined as

(13) $$\mathrm{shrink}\left(x,\sigma \right)=\left\{\begin{array}{c} x-\sigma ,\;\;x\in \left(\sigma ,\infty \right)\\ \begin{array}{c} 0,\;\;x\in \left(-\sigma ,\sigma \right)\\ x+\sigma ,\;\;x\in \left(-\infty ,-\sigma \right) \end{array} \end{array}\right.$$

for σ ≥0.

We set the number of split Bregman denoising loops as 10. After the reconstructed VMAT-CT $\left(f\right)$ was denoised by the TV-BM3D, it would be checked with the stopping criteria: if the iteration reached the maximum iteration number ($N_{\mathrm{stop}}$), or if the square difference of reconstructions between two successive iterations was below a predetermined threshold. We defined a normalized update parameter $r^{j}$ as the quantitative value for stable stopping execution:

(14) $$r^{j}=\frac{\left\|f^{j+1}-f^{j}\right\|}{\left\|f^{j=1}\right\|},\;\;for\;1<j<N_{stop}$$

We set the maximum iteration number $N_{\mathrm{stop}}$ as 20 and the threshold value for the normalized image update parameter $r^{j}$ as 0.005 based on our trials such that the change between two successive iterations becomes inappreciable. Finally, if none of the stopping criteria were met, VMAT-CT $f^{j}$ would be sent back for the local-filtered Randon transform ${\left(Rf^{j}\right)}_{L}$ of the next iteration loop.

In summary, the TV-BM3D algorithm can be described as follows (justification for the chosen parameter values and their impact on the performance of the framework can be found in Supplementary Materials):
TV-BM3D AlgorithmSet the values of parameters: μ=2,  δ=1,  α=1,  β=0.3,  r=0.005, $N_{stop}=20$.Preprocess raw EPID images *p* to obtain processed images $p_{L}$.Initialization: Blank VMAT-CT input $f^{0}$; blank forward project input (*R*$f^{0}$)*_L_*.Main iteration (*j* = 0, 1, 2, …). 1. Modified SART update. 1.1. Compute ${∆p}_{L}$ and update $f^{j}$ using Equation (5).  1.2. Enforce non-negative constraint. 2. TV-BM3D denoising (split Bregman loop). Initialization: $D_{x}^{0}=D_{y}^{0}=D_{z}^{0}=D_{w}^{0}=b_{x}^{0}=b_{y}^{0}=b_{z}^{0}=b_{w}^{0}=0;$
*f*^0^ = *f^j^*. For *k* = 0, 1, 2, … until convergence:  2.1. Update Bregman variables $D_{x}^{k+1},\;D_{y}^{k+1},D_{z}^{k+1}{,\;D}_{w}^{k+1}$ using Equation (12);  2.2. Update Bregman variables $b_{x}^{k+1},\;b_{y}^{k+1},b_{z}^{k+1}{,\;b}_{w}^{k+1}$ using Equation (10);  2.3. Update $f^{k+1}$ with the variables $D_{x}^{k},\;D_{y}^{k},D_{z}^{k}{,\;D}_{w}^{k}\;and\;b_{x}^{k},\;b_{y}^{k},b_{z}^{k}{,\;b}_{w}^{k}$ using Equation (11); End  $f^{j+1}=f^{k+1}$. 3. Stopping criterion. if $r^{j}=\frac{\left\|f^{j+1}-f^{j}\right\|}{\left\|f^{1}\right\|}$ < *r*, or $j\geq N_{stop}$. 4. Prepare for next iteration. Compute local-filtered Randon transform ${\left(Rf^{j+1}\right)}_{L}$, and return to step 1.

To accelerate the computation, we implemented the GPU-accelerated CUDA code of the forward and backward projection operators from the TIGRE toolbox version 3.1 [44] and the MEX code for the BM3D operator from the BM3D MATLAB package version 2.01 [28,45]. The computations were performed on a Dell workstation (Dell Technologies Inc., Round Rock, TX, USA) featuring an Intel Core i9-12900K 3.2 GHz CPU (Intel Corporation, Santa Clara, CA, USA), 128 GB of RAM, and a NVIDIA RTX A6000 GPU (NVIDIA Corporation, Santa Clara, CA, USA).

### 2.2. Image Quality (IQ) Analysis

We used the contrast-to-noise ratio (CNR) and structural similarity index measure (SSIM) to quantitatively evaluate the image quality of VMAT-CT.

CNR is defined as [37]

(15) $$\mathrm{CNR}=\frac{\left|{\bar{x}}_{VOI}-{\bar{x}}_{ref}\right|}{\sigma_{ref}}$$

where ${\bar{x}}_{VOI}$ is the mean voxel value within volume of interest (VOI) in VMAT-CT, $\sigma_{ref}$ is the standard deviation of the voxel value within the reference volume, and ${\bar{x}}_{ref}$ is the mean voxel value in the reference volume. The reference volume was drawn as a box in the soft tissue area, and the VOI was drawn as a box in the air cavity or bony area if no air cavity was available. Both volumes were approximately 1 cm^3^. In this study, VMAT-CT reconstruction was considered successful if CNR was higher than 2.

SSIM is defined as [46]

(16) $$SSIM=\frac{\left(2\mu_{i}\mu_{\mathrm{pCT}}+C_{1}\right)\left(2\sigma_{i}\sigma_{\mathrm{pCT}}+C_{2}\right)}{\left(\mu_{i}^{2}+\mu_{\mathrm{pCT}}^{2}+C_{1}\right)\left(\sigma_{i}^{2}+\sigma_{\mathrm{pCT}}^{2}+C_{2}\right)}$$

where *μ*_i_ and *μ*_pCT_ represent the local mean pixel values of VMAT-CT and reference CT respectively, *σ*_i_ and *σ*_pCT_ denote the local standard deviation of VMAT-CT and reference CT respectively, and *C*_1_ and *C*_2_ are regularization constants to prevent numerical instability in scenarios where μ or σ values approach zero, ensuring robust metric computation.

As we discussed in our previous study, VMAT-CT is applicable to cancer sites with sufficient density differences around the target region. For the phantom study, we used the same 50 cases based on clinical VMAT plans for multiple treatment sites (left lung (LL), right lung (RL), esophagus (ESO), and head and neck (H&N)) delivered to the Rando Chest phantom (LL, RL, ESO) or the Rando Head phantom (H&N), as explained in our previous study [5]. We also acquired 17 real-patient cases with treatment sites in the thoracic regions (RL, LL, ESO). All VMAT plans had two coplanar 6 MV arcs and were delivered using an Elekta Versa HD linac (Elekta Oncology Systems, Crawley, UK). The arc range, which is the angular span of gantry rotation—defined by the start and stop gantry angles—over which radiation is delivered continuously during a VMAT arc, and numbers of EPID images for all cases are listed in Table 1. All EPID data were recorded using the Elekta iVew system at 4 frames/second. The EPID panel has 0.8 mm × 0.8 mm pixel size and is at 160 cm source to detector distance. The number of reconstructions matches the number of cases, with each VMAT-CT dataset reconstructed into a 3D volume measuring 270 × 270 × 263 mm^3^ and an isotropic voxel size of 1 mm^3^

We used R programing language for the one-way ANOVA with split-plot design to analyze if the difference in CNR or SSIM is significant (*p* < 0.05) among VMAT-CTs reconstructed with the original method [4] (FDK-based algorithm; EPID images were processed with constant extrapolation, uniform edge erosion, and collimator angle correction) denoted as “FDK”, reconstructed with the FDK algorithm together with the systematic preprocessing methods developed by our group [5] denoted as “FDK + preprocessing”, and reconstructed with the TV-BM3D iterative algorithm together with EPID preprocessing denoted as “iterative + preprocessing”. More specifically, we compared FDK with FDK + preprocessing, FDK with iterative + preprocessing, and FDK + preprocessing with iterative + preprocessing. We used the pairwise post hoc Tukey test sequentially when ANOVA showed that the difference was significant.

## 3. Results

**Figure 2:**
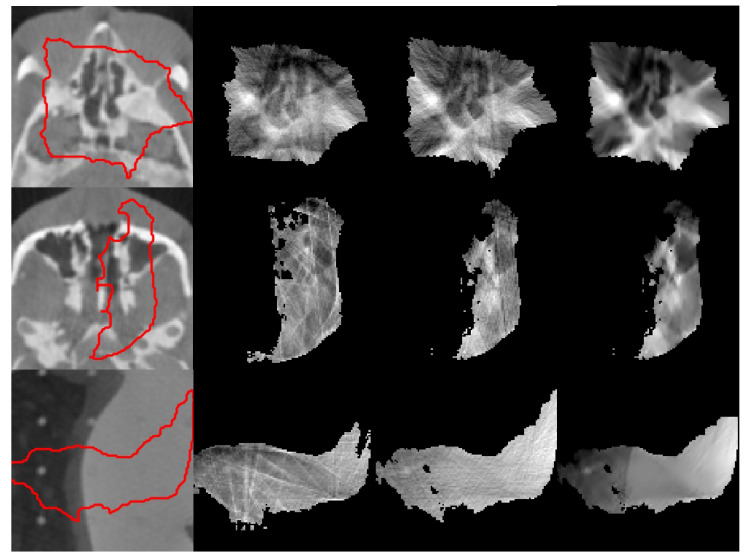
VMAT-CT reconstructions of a Rando phantom. (First column) Pretreatment CBCT overlaid by the prescription isodose lines (red); (second column) VMAT-CT reconstructed with FDK; (third column) VMAT-CT reconstructed with FDK + preprocessing; (fourth column) VMAT-CT reconstructed with iterative + preprocessing.

Figure 2 shows pretreatment CBCT and VMAT-CT images of three phantom cases. The red contour overlaid on each CBCT image corresponds to the prescription isodose line from the planning CT, transferred via rigid registration between the planning CT and CBCT. VMAT-CT images reconstructed with FDK are degraded by lots of artifacts and certain anatomy features in them are not recognizable. VMAT-CT images reconstructed with FDK + preprocessing have improved image quality, but the structures in the VMAT-CT are still not fully distinguishable because of artifacts and distortions. VMAT-CT images reconstructed with iterative + preprocessing have recognizable structures and the fewest artifacts.

**Figure 3:**
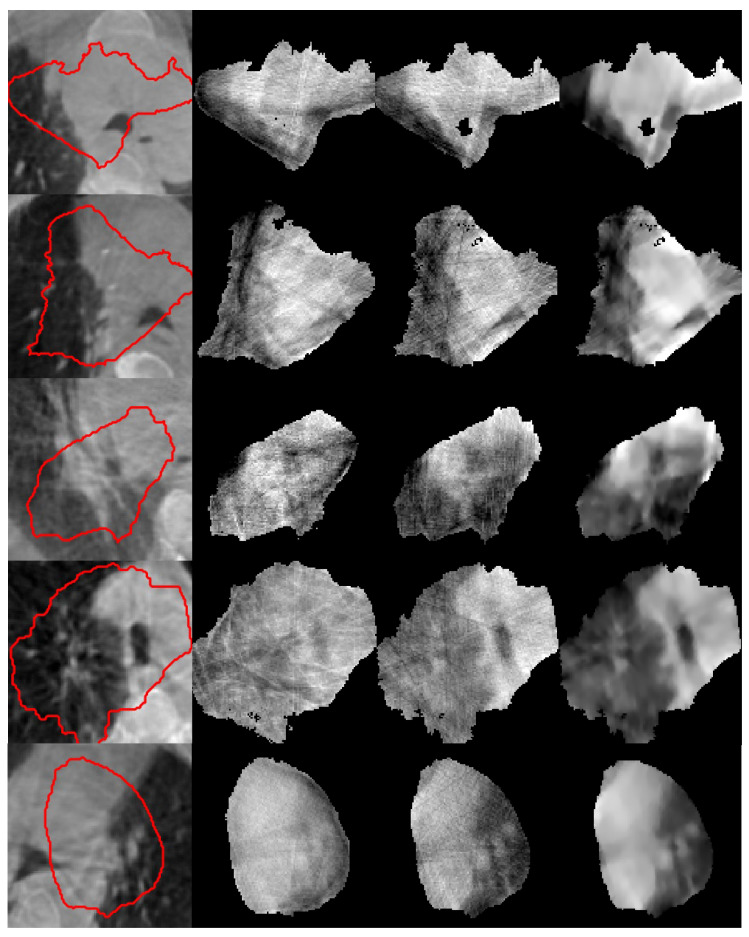
VMAT-CT reconstructions of real-patient cases. (First column) Pretreatment CBCT images overlaid by the prescription isodose lines (red); (second column) VMAT-CT reconstructed with FDK; (third column) VMAT-CT reconstructed with FDK + preprocessing; (fourth column) VMAT-CT reconstructed with iterative + preprocessing.

Similarly, Figure 3 shows five real-patient cases. VMAT-CT images reconstructed with FDK suffer from significant streaking artifacts. VMAT-CT images reconstructed with FDK + preprocessing have limited improvements. The VMAT-CT images reconstructed with iterative + preprocessing have further improved image quality and discernable anatomy structures.

In both Figure 2 and Figure 3, some reconstructions were severely affected by the angular data insufficiency and have black holes in them, which could not be resolved with the iterative algorithm. Additional challenging cases are provided in the supplementary material, further illustrating that our iterative algorithm successfully reconstructs VMAT-CT while the FDK algorithms could not.

**Figure 4:**
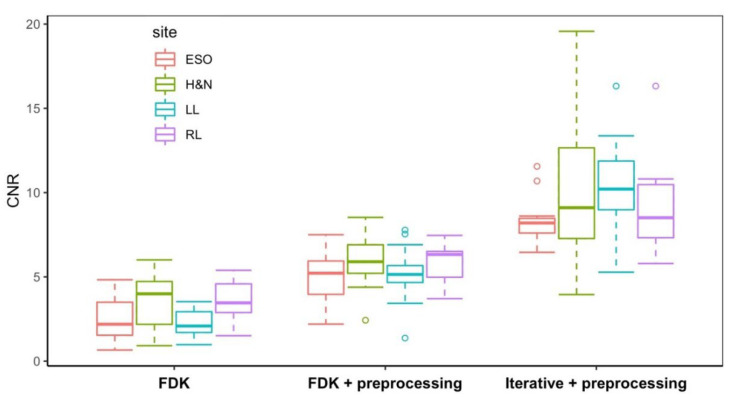
Boxplot of CNR of VMAT-CT in the phantom study.

**Figure 5:**
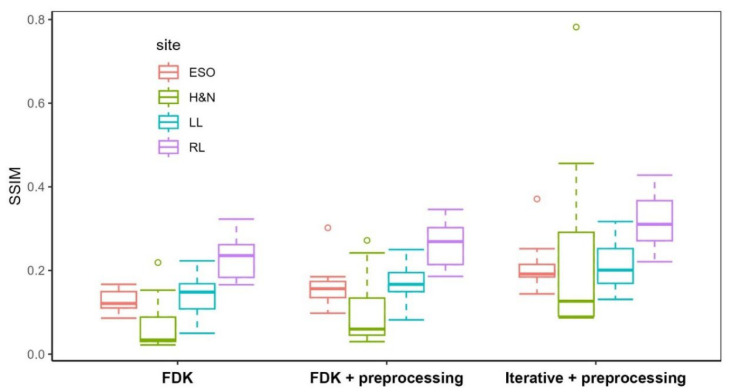
Boxplot of SSIM of VMAT-CT in the phantom study.

Figure 4 and Figure 5 present box-and-whisker plots (boxplots) of CNR and SSIM metrics for the phantom study. The central line within each box represents the median, while the lower and upper boundaries of the box correspond to the first (Q1) and third (Q3) quartiles, respectively, defining the interquartile range (IQR) that contains the middle 50% of the observations. The whiskers extend to the most extreme values within 1.5 × IQR from the quartiles, and values beyond this range are plotted as outliers in circles. These plots demonstrate how reconstruction algorithms influence VMAT-CT image quality, especially the effect of the iterative algorithm.

The post hoc Tukey tests (Table 2 and Table 3) show that combining the preprocessing method with the iterative algorithm produced statistically significant enhancements for both CNR (*p* < 0.0001) and SSIM (*p* < 0.0001).

**Figure 6:**
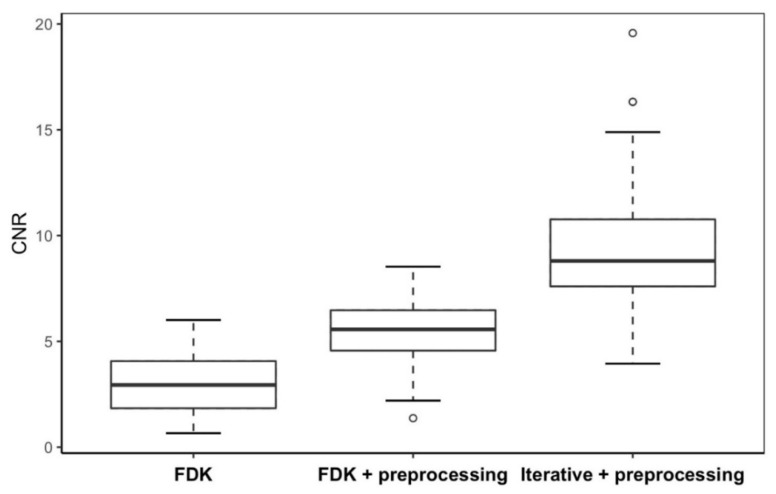
Boxplot of CNR of VMAT-CT in the real-patient study.

**Figure 7:**
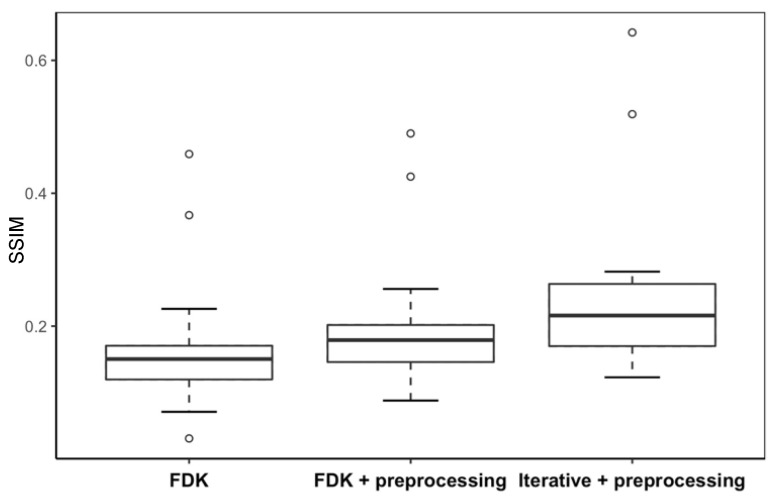
Boxplot of SSIM of VMAT-CT in the real-patient study.

Figure 6 and Figure 7 display boxplots of CNR and SSIM for real-patient cases. Both CNR and SSIM exhibit statistically significant differences (*p* < 0.0001) when different reconstruction algorithms were used.

The post hoc Tukey test (Table 4) further demonstrates that the preprocessing method and iterative algorithm each significantly enhance VMAT-CT image quality in patient cases.

## 4. Discussion

The concept of VMAT-CT was proposed a decade ago but did not gain popularity due to multiple limitations and technical challenges. Because the daily portal images during VMAT are highly blurred due to beam modulation, and commercial software cannot be used to reconstruct CT based on these images, most clinics in the US do not collect or utilize these images to our knowledge. A huge amount of image data that does not require any additional hardware, beam time or imaging dose could have been used for treatment monitoring and dose tracking purposes. There are some studies that investigated prostate localization during VMAT based on fiducial markers and portal images collected during VMAT [47,48], but this type of tracking cannot reveal patient anatomy or dose information.

In this study, we adopted the concept of CS theory, introduced TV and BM3D as the regularization constraints, and developed a TV-BM3D iterative reconstruction algorithm to improve the image quality of VMAT-CT. We succeeded in reconstructing 48 out of 50 phantom cases and 15 out of 17 patient cases using iterative + preprocessing. In contrast, only 39 phantom cases and eight patient cases could be reconstructed with FDK, and 44 phantom cases and 11 patient cases could be reconstructed with FDK + preprocessing. All phantom and patient cases show improvements in the image quality using the TV-BM3D iterative reconstruction algorithm. Our iterative algorithm can remove the irregular artifacts due to insufficient projection data and show the hidden structures in VMAT-CT that could not be revealed by the FDK-based algorithm.

The BM3D denoising algorithm characterizes pattern searching by extracting similar blocks within an image and grouping them into a few templates. With the collaborative filtering to enhance the similarity between blocks in each template, BM3D can reconstruct structures such as bones based on the assumption that these structures feature similar appearance in a medical image. On the other hand, TV minimization assumes that the voxel values within a structure in a CT image are nearly the same. Therefore, BM3D and TV exploit data sparsity with different assumptions, and both provide constraints for the iterative algorithm to solve the sparse data problem in CT reconstruction, making the TV-BM3D iterative algorithm more effective than TV minimization or BM3D alone. The proper choice of block size and noise level in the BM3D method is crucial for the denoising performance, and extra tuning efforts are required to balance the denoising power of TV and BM3D regularizations.

Reconstruction from incomplete projection data, such as limited-angle or truncated field-of-view CT, is an ill-posed problem in which analytical algorithms like the FDK often produce strong streak artifacts and noise amplification due to violation of the full-sampling assumption. Iterative reconstruction methods incorporating sparsity constraints have therefore become the state-of-the-art for sparse or truncated CT data. In particular, TV regularization has been widely used to suppress streak artifacts and stabilize reconstruction from limited projections, although it may introduce over-smoothing and loss of fine anatomical details, particularly under severe data incompleteness [18]. Several studies have proposed improved regularization models to overcome these limitations. For example, compressed-sensing-based CT reconstruction frameworks demonstrated that sparse regularization could significantly improve image quality with reduced projection data, forming the theoretical foundation for many modern iterative CT reconstruction algorithms. Subsequent developments introduced adaptive or relative TV models to better preserve edges and textures under limited-angle acquisition. More advanced methods incorporate additional priors such as non-local patch similarity or prior-image constraints to better preserve structural information [49]. For example, the prior-image-constrained compressed sensing framework and non-local regularization approaches have demonstrated improved reconstruction accuracy for undersampled CT data [50].

Building on these developments, the proposed algorithm integrates local sparsity constraints (TV) with non-local self-similarity priors (BM3D) within a unified optimization framework solved using the Split Bregman method [28,39]. This hybrid regularization strategy improves both noise suppression and structural preservation compared with analytical reconstruction and TV-only methods. Quantitatively, the proposed method achieved CNR values ranging from 3.61 to 19.57 (mean ≈ 9.3) and SSIM values ranging from 0.087 to 0.782 (mean ≈ 0.27) across all 64 cases. While the mean SSIM appears lower than some reports in the literature, meaningful comparison requires careful consideration of acquisition conditions, reference definitions, and task difficulty, particularly in sparse-view and limited-angle CT reconstruction.

TV-based reconstruction remains a standard baseline for sparse-view and limited-angle CT. Conventional TV-based baselines have demonstrated SSIM values of approximately 0.812–0.960 and CNR values of 1.97–7.27 under sparse-view conditions (30–90 projections) on the Shepp–Logan phantom. More advanced TV-based variants, such as reinforced TV (rTV), have pushed performance further with SSIM up to 0.984 and CNR up to 14.26 under 90-projection sparse-view acquisition [51]. In more demanding limited-angle configurations, single-energy TV regularization has yielded SSIM ≈ 0.88 and CNR ≈ 2.8 on anthropomorphic phantoms [52]. More recent work, such as Xi et al. [53], reports SSIM values of approximately 0.85–0.93 for standard TV and up to ~0.90–0.97 for advanced high-order TV formulations, when evaluated against matched full-view reference images under moderate sparse-view conditions. However, these high SSIM values are largely attributable to matched-reference evaluation and moderate undersampling regimes. Importantly, TV-based methods inherently impose piecewise-constant assumptions, which suppress noise but also attenuate low-contrast features and fine textures, leading to moderate CNR improvement but reduced structural fidelity, particularly in highly undersampled scenarios.

Recent studies have explored deep learning-based reconstruction or sinogram completion for limited-angle CT. Across deep learning-based methods, SSIM values are typically reported in the range of ~0.80–0.95 under moderate sparse-view or low-dose conditions, depending on the similarity between training and testing distributions [54]. However, these methods are typically evaluated on datasets with consistent geometries, full-angular sampling ranges, and reference reconstructions from dense-view filtered back-projection. Moreover, they often rely on large, well-matched training datasets, supervised learning with high-quality ground truth, and limited generalizability across imaging systems or treatment sites [55]. In contrast, the proposed approach operates directly on measured portal images without the need for training data, supporting the feasibility of VMAT-CT as a practical in-treatment imaging modality for treatment monitoring and adaptive radiotherapy.

What fundamentally distinguishes our study from the vast majority of the sparse-view and limited-angle CT reconstruction literature is the nature of the raw data. Nearly all benchmark studies—including those employing TV-based methods, advanced TV variants, and deep learning approaches—evaluate performance on kV CT datasets acquired under idealized or controlled conditions: well-calibrated geometries, consistent photon flux, and relatively predictable noise characteristics. In these studies, the primary ill-posedness stems solely from angular undersampling or restricted scan ranges, with the underlying projection data remaining otherwise coherent and physically well-behaved. In stark contrast, our data originate from MV portal imaging, which introduces a cascade of compounded degradations absent from conventional benchmarks: inherently poor image quality due to high-energy photon physics, severe irregularity from MLC modulation that creates highly non-uniform fluence patterns, substantial blurring from both MLC motion during delivery and scatter-dominated signals, and extreme angular incompleteness far beyond typical limited-angle scenarios. Consequently, where standard sparse-view studies address reconstruction under ideal acquisition models with controlled subsampling, we confront a regime in which the forward model itself is corrupted by time-varying modulation, mechanical motion, and physical degradations that violate nearly all conventional assumptions. This places our reconstruction task in a fundamentally more challenging class of problems, rendering direct quantitative comparisons of CNR and SSIM across studies inherently inequitable without careful contextualization of the underlying data fidelity and acquisition physics. That being said, the proposed method demonstrates substantial performance gains, with multiple cases achieving CNR > 14–19, exceeding the typical upper range reported for conventional iterative reconstruction. Similarly, the upper range of SSIM values (0.4–0.8) approaches or surpasses those observed in early deep learning-based reconstruction frameworks. To our knowledge, this work represents the first demonstration of an iterative reconstruction framework specifically designed for VMAT-CT, and it highlights the potential of advanced regularization methods to overcome the severe data incompleteness inherent in treatment-time imaging.

There are several limitations of this study. First, the stopping threshold value (0.005) is determined by our trials of VMAT-CT reconstruction. However, the convergence is affected by the strengths of BM3D denoising which is tuned by the noise levels in BM3D, as well as TV minimization which is tuned by steepest-descent step size and the number of steps within the inner-iteration. If the regularizations of BM3D denoising and TV minimization are adjusted unbalanced, the VMAT-CT may be overly smoothed at each iteration step such that the updated parameter $r^{j}$, which represents the change between successive VMAT-CT iterations, will be too large to converge. Second, some failed cases of VMAT-CT remain unsolved even with the iterative algorithm. For VMAT-CT with extremely poor quality, tuning the proper block size and finding the patterns for BM3D could be challenging and correct templates could not be represented by blocks. One feasible approach is that the regularization models in our algorithm could be decomposed and replaced by a convolutional neural network (CNN) such that the limitations of tuning regularization parameters of TV and BM3D can be relieved. For example, some groups introduced deep CNN into the alternating direction method of multipliers (ADMMs) iterative reconstruction algorithm as the regularization term to solve the distorted limited-angle CT images, and found better image recovery than the iterative algorithms with TV regularization [56,57]. Finally, the speed of the iterative algorithm is relatively slower. Table 5 shows the overall computational time of the whole 3D VMAT-CT reconstruction. Compared with FDK and FDK + preprocessing, iterative + preprocessing takes the longest time ranging between 6 and 10 min because of the varying convergence speed for different cases of VMAT-CT. The computational bottleneck of the iterative reconstruction is the BM3D denoising, which involves computationally demanding processes such as block-matching, grouping, and aggregation. There are several studies in the literature about GPU-based BM3D denoising, but they are limited to applications to a 2D image and require memory organization and thread cooperation for data exchange [58]. Future work on 3D GPU-based BM3D denoising in MATLAB could further accelerate the TV-BM3D iterative algorithm.

In the future, the framework of our TV-BM3D iterative algorithm could be revised to have faster convergence and require less tuning. Instead of optimizing in two alternative phases, the optimization problem may be solved using Barzilai–Borwein formulation in a single phase [7]. Because the tuning parameters, including regularization weighting factor, block size and noise levels of BM3D, step size and iteration number of TV minimization, are affected by some characteristics of VMAT plans such as MLC modulation complexity score [59], small aperture score for the aperture size [60], and the inherit CT contrasts at the locations of treatment sites [61], we can reduce the tuning labors in the clinical workflow by pre-setting these parameters as specific protocols for each treatment site, which is similar to the kV-CBCT protocols used in the clinic.

## 5. Conclusions

A TV-BM3D iterative reconstruction algorithm was proposed for VMAT-CT reconstruction. This algorithm significantly outperformed traditional methods: while at most 44/50 phantom cases and 11/17 real-patient cases could be reconstructed with the FDK algorithms, the proposed iterative method successfully reconstructed 48/50 phantom cases and 15/17 real-patient cases. This represents a substantial improvement in reconstruction success rate. Moreover, our algorithm significantly enhances the image quality of VMAT-CT across all treatment sites. To our knowledge, this is the first iterative reconstruction algorithm developed specifically for VMAT-CT. This study, together with our previous work [4,5,62], strongly supports VMAT-CT as a promising 3D and four-dimensional (4D) imaging tool for treatment monitoring and adaptive RT.

## Figures and Tables

**Table 1:** Arc ranges and number of acquired EPID images per treatment site.

	ESO	LL	RL	H&N
Arc range	[−175, 175]	[−30, 175]	[−175, 30]	[−175, 175]
Number of EPID images	281 ± 127	213 ± 67	229 ± 48	248 ± 86

**Table 2:** The post hoc Tukey test of CNR of VMAT-CT in the phantom study for various combinations of reconstruction methods and treatment sites.

	CNR (*p* Value)			
	ESO	LL	RL	H&N
FDK vs. FDK + preprocessing	<0.0001	0.0004	0.0117	0.0368
FDK vs. iterative + preprocessing	<0.0001	<0.0001	<0.0001	<0.0001
FDK + preprocessing vs. iterative + preprocessing	<0.0001	<0.0001	0.0001	0.0002

**Table 3:** The post hoc Tukey test of SSIM of VMAT-CT in the phantom study for various combinations of reconstruction methods and treatment sites.

	SSIM (*p* Value)			
	ESO	LL	RL	H&N
FDK vs. FDK + preprocessing	0.0102	0.0356	0.0037	0.8084
FDK vs. iterative + preprocessing	0.0013	0.0002	<0.0001	0.0473
FDK + preprocessing vs. iterative + preprocessing	<0.0001	<0.0001	<0.0001	0.012

**Table 4:** The post hoc Tukey test in CNR and SSIM of VMAT-CT in the real-patient study for various combinations of reconstruction methods, using post hoc Tukey tests.

	CNR (*p* Value)	SSIM (*p* Value)
FDK vs. FDK + preprocessing	0.0088	0.0339
FDK vs. iterative + preprocessing	<0.0001	<0.0001
FDK + preprocessing vs. iterative + preprocessing	0.0002	0.0008

**Table 5:** Computational time of VMAT-CT reconstructions.

Algorithm	Time (s)
FDK	153 ± 61
FDK + preprocessing	307 ± 91
Iterative + preprocessing	471 ± 122

## Data Availability

The original contributions presented in this study are included in the article and Supplementary Material. Further inquiries can be directed to the corresponding author.

## Supplementary Materials

The following supporting information can be downloaded at: https://www.mdpi.com/article/10.3390/jimaging12040166/s1, Figure S1: Our framework demonstrates successful reconstructions of VMAT-CT for challenging cases, in contrast to FDK algorithm that cannot perform such reconstructions. (First column) Pretreatment CBCT overlaid by the prescription isodose lines (red); (second column) VMAT-CT reconstructed with FDK; (third column) VMAT-CT reconstructed with FDK + preprocessing; (fourth column) VMAT-CT reconstructed with iterative + preprocessing. Refs. [63,64,65,66,67,68] are cited in Supplementary Materials.
