# Supplementary material for: A TV–BM3D Iterative Algorithm for VMAT-CT Reconstruction
Source: J Imaging. 2026 Apr 10;12(4):166. doi: 10.3390/jimaging12040166 (PMC13118252; doi:10.3390/jimaging12040166)
Supplement: Supplementary file 1 [file jimaging-12-00166-s001.zip › jimaging-4166931-supplementary.pdf]

Supplementary Materials

Justification for chosen parameters´ values and how their values affect the performance of our framework.

The data fidelity weight μ controls the balance between projection consistency and prior regularization. In iterative CT reconstruction, μ is typically selected within the range $\mu \approx 0.5–5$ depending on noise level and sampling sparsity to balance data fidelity and regularization strength [18,63]. Values below this range lead to over-regularization, characterized by loss of anatomical contrast and piecewise-constant ("cartoon-like") appearance dominated by total variation (TV), while excessively large μ amplifies noise and streak artifacts due to sparse VMAT projections. The chosen value $\mu = 2$ lies within this recommended range and was found to provide a stable balance between data fidelity and prior-driven regularization.

The parameter δ was selected to maintain comparable weighting between the TV and BM3D priors, consistent with hybrid regularization frameworks in which local and non-local constraints are balanced to jointly preserve edges and recover nonlocal image textures [28,64]. Smaller δ leads to TV-dominant reconstructions with staircase artifacts, whereas larger δ results in over-smoothed or patch-corrupted textures. The selected value $\delta = 1$ lies centrally within this range (0.5 – 2) and ensures complementary regularization behavior.

Within the split Bregman framework (split Bregman method), the parameters α and β control the enforcement strength of the auxiliary constraints associated with TV and BM3D terms, respectively. Empirically, α is typically chosen in the range 0.5–2 to ensure stable convergence without numerical stiffness, while β is often selected as $\beta \approx 0.1–1$ and generally smaller than α to avoid over-dominance of the nonlocal prior [39,65]. In this work, $\alpha = 1$ and $\beta = 0.3$ were adopted, both well within these ranges, providing stable convergence and balanced regularization.

Convergence parameters were selected to ensure computational efficiency without com-promising solution accuracy. The stopping tolerance r is commonly set between 10^{-2} and 10^{-4} in iterative reconstruction algorithms; larger values risk premature termination, whereas smaller values yield diminishing returns in image quality at increased computational cost. We selected $r = 5\times10^{-3}$, which falls within this practical range. The maximum iteration number N_stop is typically chosen between 10 and 50 for split Bregman-based CT reconstruction [39,66]; here, N_stop = 20 was selected based on convergence experiments, achieving a favorable trade-off between runtime and reconstruction quality.

For BM3D-specific parameters, the block (patch) size and search step directly affect the nonlocal prior. A patch size of 8×8 is widely regarded as optimal for balancing noise sup-pression and detail preservation, compared to smaller (4×4) patches that are noise-sensitive and larger (16×16) patches that risk over-smoothing [28,67]. The patch step (sliding stride) is typically chosen between 1 and 3 pixels; smaller steps improve de-noising performance at increased computational cost, while larger steps reduce redundancy and degrade performance. In this work, the default BM3D implementation settings (step = 3) were used, consistent with prior studies.

The hard-threshold parameter λ in BM3D is theoretically proportional to the noise standard deviation ($\lambda \approx 2.7$–3.0σ) in the transform domain [28]. However, in the MATLAB reference implementation, both image intensities and noise levels are normalized to [0,1], and λ is internally calibrated relative to this normalization and transform-domain statistics rather than directly to physical units [67]. Accordingly, $\lambda = 1.5$ (default value) was used, which lies within the empirically validated range $\lambda \approx 1.0$–2.5 for normalized images. Sensitivity analysis confirmed that smaller values resulted in residual noise, while larger values caused excessive smoothing.

The noise variance parameter σ^2 defines the assumed noise level. For CT imaging, $\sigma \approx 15$–25 HU is commonly reported [68]. After normalization to [0,1], this corresponds to $\sigma \approx 0.005$–0.02 depending on the intensity range. The selected value $\sigma^2 = 1\times10^{-4}$ ($\sigma = 0.01$) falls squarely within this normalized range and is consistent with effective noise levels (~20 HU) observed in VMAT-CT, where noise is inherently spatially varying and projection-dependent.

Finally, regarding the step size in TV minimization, it is important to note that in the split Bregman formulation, the optimization does not rely on an explicit gradient descent step size. Instead, the parameters α and β implicitly control the update magnitude and convergence behavior of the TV subproblem [39]. Therefore, the “step size” is effectively governed by these parameters rather than an explicit tuning variable.

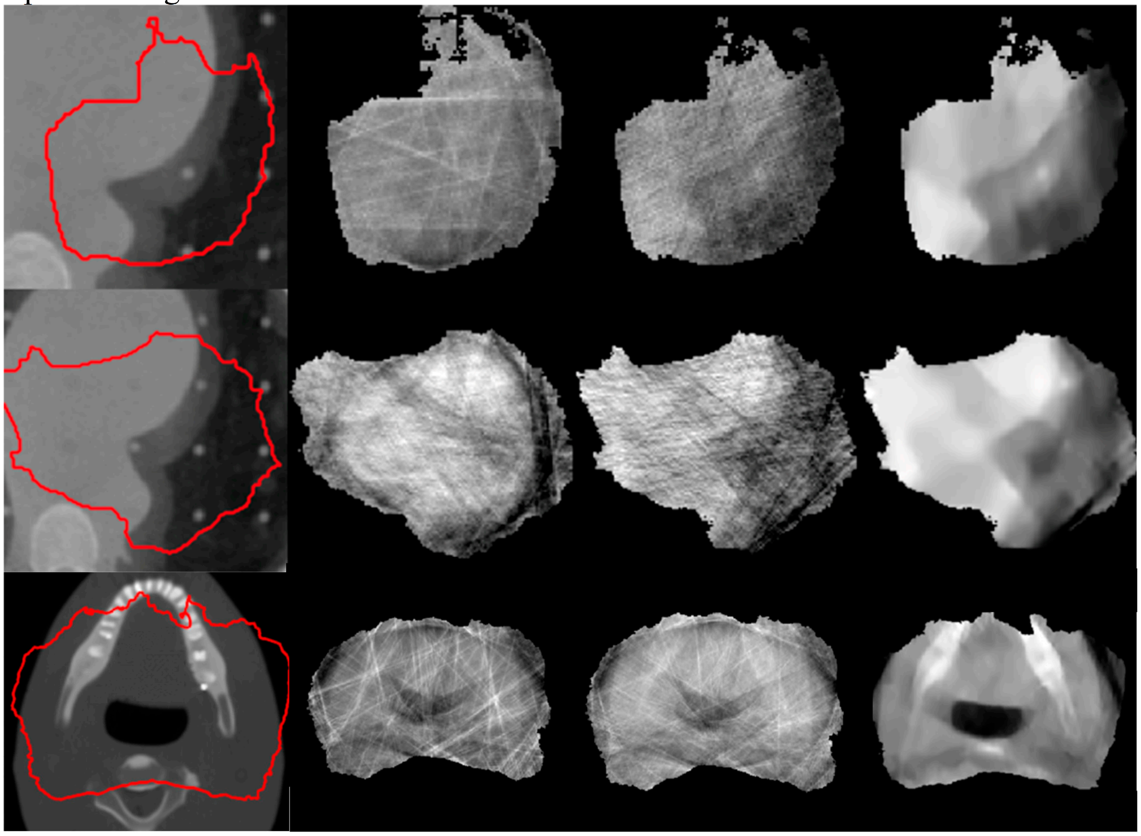

Suppl. Figure S1. Our framework demonstrates successful reconstructions of VMAT-CT for challenging cases, in contrast to the FDK algorithm that cannot perform such reconstructions. (First column) Pretreatment CBCT overlaid by the prescription isodose lines (red); (second

column) VMAT-CT reconstructed with FDK; (third column) VMAT-CT reconstructed with FDK + preprocessing; (fourth column) VMAT-CT reconstructed with iterative + preprocessing.
